# Using Serum Advanced Glycation End Products-Peptides to Improve the Efficacy of World Health Organization Fasting Plasma Glucose Criterion in Screening for Diabetes in High-Risk Chinese Subjects

**DOI:** 10.1371/journal.pone.0137756

**Published:** 2015-09-14

**Authors:** Zilin Sun, Jiajia He, Shanhu Qiu, Chenghao Lei, Yi Zhou, Zuolin Xie, Lin Zhang, Yanping Wang, Hui Jin

**Affiliations:** 1 Department of Endocrinology, Zhongda Hospital, Southeast University, Nanjing, P.R. China; 2 Institute of Diabetes, School of Medicine, Southeast University, Nanjing, P.R. China; Baylor College of Medicine, UNITED STATES

## Abstract

The efficacy of using fasting plasma glucose (FPG) alone as a preferred screening test for diabetes has been questioned. This study was aimed to evaluate whether the use of serum advanced glycation end products-peptides (sAGEP) would help to improve the efficacy of FPG in diabetes screening among high-risk Chinese subjects with FPG <7.0 mmol/L. FPG, 2-h plasma glucose (2h-PG), serum glycated haemoglobin A1c (HbA1c), and sAGEP were measured in 857 Chinese subjects with risk factors for diabetes. The areas under receiver operating characteristic (ROC) curves generated by logistic regression models were assessed and compared to find the best model for diabetes screening in subjects with FPG <7.0 mmol/L. The optimal critical line was determined by maximizing the sum of sensitivity and specificity. Among the enrolled subjects, 730 of them had FPG <7.0 mmol/L, and only 41.7% new diabetes cases were identified using the 1999 World Health Organization FPG criterion (FPG ≥7.0 mmol/L). The area under ROC curves generated by the model on FPG-sAGEP was the largest compared with that on FPG-HbA1c, sAGEP, HbA1c or FPG in subjects with FPG <7.0 mmol/L. By maximizing the sum of sensitivity and specificity, the optimal critical line was determined as 0.69×FPG + 0.14×sAGEP = 7.03, giving a critical sensitivity of 91.2% in detecting 2h-PG ≥11.1 mmol/L, which was significantly higher than that of FPG-HbA1c or HbA1c. The model on FPG-sAGEP improves the efficacy of using FPG alone in detecting diabetes among high-risk Chinese subjects with FPG <7.0 mmol/L, and is worth being promoted for future diabetes screening.

## Introduction

Diabetes mellitus has become a widespread epidemic. The prevalence of diabetes has rapidly increased from 9.7% in 2007 to 11.6% in 2010 among Chinese population [1,2]. Notably, the latest national survey from China has shown that up to 70% of the people with diabetes are left undiagnosed [2], and accumulating evidence has noted that diabetes-associated complications are sometimes already present in those newly diagnosed patients [3–5]. Early detection of diabetes could enable timely therapeutic or lifestyle interventions to prevent these complications, as well as to reduce the related death rates [6–9]. These facts therefore greatly support the critical need to detect individuals with undiagnosed diabetes or those who are at risk of diabetes in a timely and efficient fashion.

Fasting plasma glucose (FPG) and oral glucose tolerance test (OGTT) have been highly recommended to screen asymptomatic individuals. Although FPG is easy to access and reproducible [10], increasing evidence has raised concerns regarding its efficacy as a single test in diabetes screening, since a considerable proportion of diabetes patients that have normal FPG but elevated 2-h plasma glucose (2h-PG) might be undetected [11–13]. OGTT is considered the gold standard in the diagnosis of diabetes, and is especially recommended for high-risk individuals who have FPG <7.0 mmol/L. However, it is worth noting that it might be difficult and impractical to get all individuals to use OGTT due to its complexity, inconvenience and low response rate [14]. Moreover, current guidelines have not specified a screening or diagnostic strategy for this highly at-risk group.

Glycated haemoglobin A1c (HbA1c) reflects the average blood glucose concentration during the preceding 8–12 weeks [15], which has been suggested as an promising approach for diabetes screening or diagnosing [16,17]. Previous studies have shown that HbA1c could significantly increase the efficacy of using FPG alone in detecting diabetes among individuals with FPG <7.0 mmol/L [17,18]. However, the lack of standardization in HbA1c assay and the relatively expensive cost largely limits its use for screening in Chinese population [19,20].

Advanced glycation end products (AGEs) are formed from the nonenzymatic or Maillard reactions between reducing sugars and the free amino groups of proteins or amino acids, and their accumulation is increased or accelerated when exposed to long-term hyperglycemia (e.g., diabetes) [21]. AGE-peptides (AGEP) are generated from AGEs [22]. These small-sized peptides exhibit the same toxic activity of AGEs to the tissues of blood vessels [23,24], and have been recognized responsible for the tissue modification observed in the complications of diabetes (due to their higher production) such as diabetic nephropathy [25,26]. Moreover, previous work noted that AGEP showed more marked differences between patients with diabetes and healthy controls than did AGEs [27]. These indicate that AGEP are potential biomarkers for diabetes and might be more sensitive than AGEs in detecting diabetes. Furthermore, the measurement assay for determining AGEP concentrations in serum has been successfully validated in previous studies, which is reliable and reproducible [22,28,29]. In order to provide an alternative way to improve diabetes screening, this exploratory study was aimed to examine whether the use of serum AGEP (sAGEP) would help to improve the efficacy of using FPG alone in diabetes screening, as well as to determine the respective optimal critical line for detecting diabetes among high-risk Chinese subjects with FPG <7.0 mmol/L.

## Materials and Methods

### Study design and population

This cross-sectional survey [29] was conducted in 9 community health centers in Nanjing, China, and begun from September in 2010 to September in 2011. Subjects were randomly chosen from those who were registered in the community health centers and aged over 18 years using notice or advertisement. Subjects were eligible for inclusion if they were considered at high-risk for diabetes according to the following criteria: (1) being diagnosed with impaired fasting glucose (IFG) or impaired glucose tolerance (IGT); (2) being older than 40 years; (3) being overweight or obese (body mass index [BMI] ≥25 kg/m^2^); (4) having a first degree relative with diabetes; (5) having a history of giving birth to a baby weighing more than 4 kg, or being diagnosed with gestational diabetes; (6) having hypertension (140/90 mmHg or above) or taking antihypertensive drugs; (7) having hyperlipidemia or taking lipid-regulating drugs; (8) having a history of heart-cerebro-vascular disease, or being physically inactive (<150 minutes/week); (9) having a history of transient hyperglycemia induced by steroid; (10) having polycystic ovary syndrome with BMI ≥30 kg/m^2^. Subjects were excluded if they were diagnosed with diabetes, cancer, severe psychiatric disturbance, or chronic kidney disease, had a history of acute kidney injury in recent one year, or failed to complete the survey.

**Fig 1:**
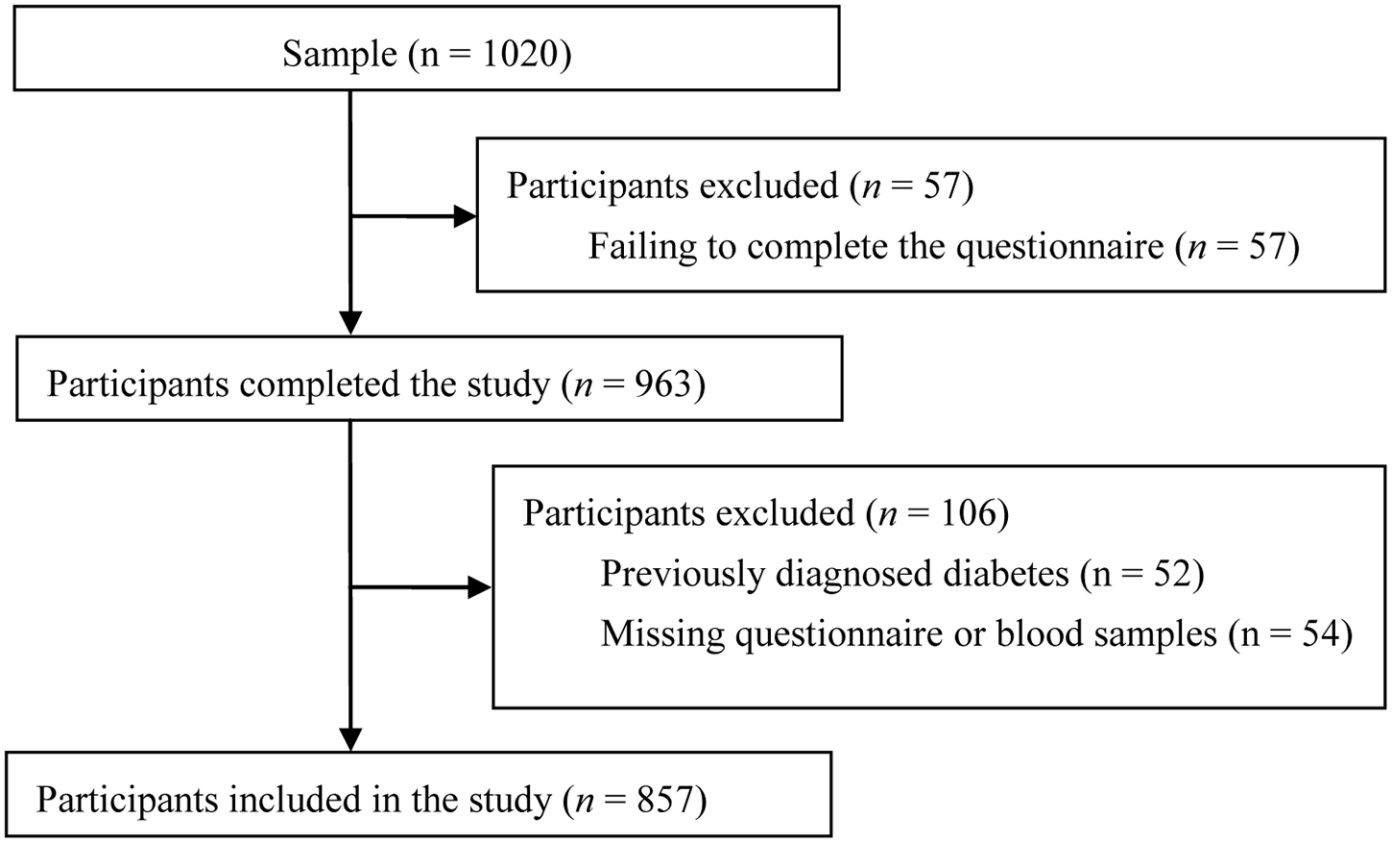
Flow diagram of the recruitment of Chinese subjects.

The average response rate was 93%. A total of 1020 Chinese people with risk factors aged from 19 to 88 years participated in the survey. Among them, 57 subjects failed to complete the pre-designed questionnaire mainly due to the lack of time. 106 subjects were further excluded, because 52 of them were already diagnosed with diabetes, and 54 had the questionnaire or blood samples missed. Therefore, 857 subjects were included in the final analyses (Fig 1). The study protocol was approved by the Medical Ethics Committee of Zhongda Hospital, Southeast University, and all subjects provided written informed consent. This study is also reported with reference to the Standards for Reporting of Diagnostic Accuracy (STARD) [30].

### Anthropometric and laboratory measurements

All eligible subjects were invited to complete a uniform questionnaire including questions about their current and past medical history, as well as medications. After an overnight fasting (at least 10 hours), the fasting venous blood samples for each subject were drawn for the measurements of FPG, HbA1c and AGEP. Afterwards, each subject had a 75 g OGTT, the second blood samples were taken 2 hours later for the measurement of 2h-PG.

The anthropometric parameters included body weight, height, and blood pressure (BP). BMI was calculated as weight (kg) divided by squared height (m). BP was the mean of 2 measurements made with a sphygmomanometer at a 2-minute interval. Plasma glucose was measured by glucose oxidase method. HbA1c was analyzed using high performance liquid chromatography (HPLC) (Bio-RAD D-10, America), with the inter- and intra-assay coefficients of variation <0.4% and <2.0%, respectively.

sAGEP were measured by an experienced technician who was blinded to the study using a modified method described previously [22,28]. Briefly, all measurements were performed with the flow injection assay assembled with HPLC (Waters 600, America). For each measurement, 20 μl of serum from each sample was mixed with 480 μl of trichloroacetic acid (0.15 mol/L) and 100 μl of chloroform in a microcentrifuge tube. The tube was vortexed thoroughly at regular intervals and then centrifuged at 13 000g for 10 minutes. A 20 μl of the aqueous layer was injected to the sample injector of HPLC. To detect the signal of AGEP, the spectrofluorometric detector was set to 440 nm for the emission wavelength and 370 nm for the excitation wavelength. The average peak height from each sample assayed in triplicate was used for analyzing. Since no AGEs standard is available at current, external calibration was conducted using an AGEP calibrator obtained by hydrolysis of calibrator AGEs-BSA (50 g/L) incubating with proteinase K (8 g/L). The unit of AGEP was expressed as U/ml, with 1.0 U/ml equating to the protein concentration obtained by degradation of 1.0 mg/L AGEs-BSA described above. A series of AGEP calibrator dilution (0.01 U/ml, 0.10 U/ml, 5.0 U/ml, 10.0 U/ml, 20.0 U/ml, and 40.0 U/ml AGEP) was prepared using the AGEs-BSA calibrator as the reference, and was used to obtain the standard curve for calculating the concentrations of AGEP in unknown samples using a linear model (S1 Fig). The within-day and between-day precision for 10 measurements of one sample during 5 days were 2.9% and 3.0%, respectively.

### Definition

Based on the 1999 World Health Organization (WHO) criteria, diabetes is defined as FPG ≥7.0 mmol/L, 2h-PG ≥11.1 mmol/L, or both. Impaired glucose regulation (IGR) is defined as IGT (FPG <6.1 mmol/L and 2h-PG ≥7.8 and <11.1 mmol/L), IFG (FPG ≥6.1 mmol/L and <7.0 mmol/L and 2h-PG <7.8 mmol/L), or IGT with IFG (FPG ≥6.1 mmol/L and <7.0 mmol/L and 2h-PG ≥7.8 mmol/L and <11.1 mmol/L). Normal glucose tolerance (NGT) is defined as FPG <6.1 mmol/L and 2h-PG <7.8 mmol/L.

### Statistical analysis

Statistical analyses were performed with reference to the handbook of biological statistics [31] using SPSS software (Version 17.0 for Windows, SPSS Inc, Chicago, IL, USA) and STATA (Version 12.0 StataCorp, College Station, TX, USA). Demographic and clinical variables were presented as means and standard deviations, and compared using the independent-samples *t* test in men and women. One-way analysis of covariance was used to compare differences in sAGEP levels among diabetic, IGR, and NGT subjects, with adjustment for age. Partial Pearson correlation analysis adjusting for age was conducted to quantify the association between sAGEP and variables regarding glycemic control (that is, FPG, 2h-PG and HbA1c). A two-tailed *P* value <0.05 was considered to be statistically significant.

Logistic regression model on FPG, HbA1c, sAGEP, FPG plus HbA1c (FPG-HbA1c) or FPG plus sAGEP (FPG-sAGEP) was separately used to generate the receiver operating characteristic (ROC) curve for assessing the ability of each model in detecting diabetes among subjects with FPG <7.0 mmol/L. The area under each ROC curve was compared by using the method described by DeLong *et al*. [32] to find the best model among the five. The model on FPG-sAGEP was later found to be the best one from our data. The optimal critical line for this model was derived from the ROC curve by maximizing the sum of sensitivity and specificity, which is as follows:

$$\text{b}\times {\text{FPG}}_{\text{i}}+\text{c}\times {\text{sAGEP}}_{\text{i}}=\text{log}\left(\frac{\text{k}}{\left[1-\text{k}\right]}\right)-\text{a}$$

It indicated that the participant i whose FPG_i_ and sAGEP_i_ were above or on the line would be suggested to take an OGTT as a confirmative test for diabetes, in which a, b, c, and k were the estimated intercept, coefficient for FPG, coefficient for sAGEP, and the optimal cutoff point determined by maximizing the sum of sensitivity and specificity, respectively. The procedures in obtaining the optimal critical line were generally referred to the method described by Wang *et al*. [17]. Comparisons among the critical sensitivities were made using the McNemar test [33].

## Results

The demographic and clinical characteristics are presented in Table 1, where there were 290 men (30.1%) and 567 women (69.9%) enrolled. The men were older and had higher systolic and diastolic BP, FPG, and 2h-PG than women (all *P* <0.01). The frequency distribution of FPG by 2h-PG for these 857 subjects based on the WHO diagnostic criteria is shown in Table 2. A total of 218 (25.4%) subjects were new cases of diabetes, 229 (26.7%) had IGR, and 410 (47.8%) had NGT. Of the 218 new diabetic subjects (94 men and 124 women), 127 had FPG ≥7.0 mmol/L, suggesting that if FPG was used alone in diabetes screening, its sensitivity would be 58.3%. Of the 180 subjects with 2h-PG ≥11.1 mmol/L (74 men and 106 women), 91(50.6%) had FPG <7.0 mmol/L, accounting for 12.5% (91/730) in this high risk population with FPG <7.0 mmol/L.

**Table 1:** General and clinical characteristics of Chinese subjects.

	*Men (n = 290)*	*Women (n = 567)*	*P* ^a^
Age(years)	62.4 ± 11.8	59.6 ± 12.0	0.001
BMI (kg/m^2^)	24.9 ± 3.3	24.4 ± 3.7	0.06
SBP (mmHg)	132.4 ± 18.5	128.9 ± 19.2	0.007
DBP (mmHg)	82.2 ± 11.3	79.5 ± 10.4	0.001
FPG (mmol/L)	6.2 ± 2.5	5.8 ± 1.5	0.008
2h-PG (mmol/L)	9.0 ± 4.1	8.3 ± 3.9	0.009
HbA1c (%)	6.3 ± 1.0	6.2 ± 1.0	0.22
sAGEP (U/ml)^b^	11.0 ± 7.3	10.0 ± 8.0	0.21^c^

BMI, body mass index; SBP, systolic blood pressure; DBP, diastolic blood pressure; FPG, fasting plasma glucose; 2h-PG, 2h-plasma glucose; HbA1c, glycated haemoglobin A1c; sAGEP, serum advanced glycation end products-peptides

Data are presented as means ± standard deviations.

^a^ Compared between men and women.

^b^Samples for measuring sAGEP were taken from 856 subjects with 290 men and 566 women.

^c^ Compared between men and women with adjustment for age.

**Table 2:** Number of subjects according to the categorization of glycemic status using FPG and 2h-PG based on the 1999 WHO diagnostic criteria.

	FPG (mmol/L)			
2h-PG (mmol/L)	<6.1	≥6.1 and <7.0	≥7.0	Total
<7.8	410	58	15	483
≥7.8 and <11.1	129	42	23	194
≥11.1	48	43	89	180
Total	587	143	127	857

FPG, fasting plasma glucose; 2h-PG, 2h-plasma glucose; WHO, World Health Organization

sAGEP were significantly higher in diabetic subjects (18.68 ± 10.88 U/ml) compared with IGR (8.73 ± 3.42 U/ml, *P* <0.001) or NGT subjects (6.78 ± 2.20 U/ml, *P* <0.001) with adjustment for age, but were comparable in men and women (*P* = 0.21, Table 1). sAGEP were significantly and directly correlated with FPG (*r* = 0.64, *P* <0.001), 2h-PG (*r* = 0.75, *P* <0.001), and HbA1c (*r* = 0.90, *P* <0.001) in the overall population. However, these correlations were weaker but significant among diabetic, IGR, or NGT subjects in general (Table 3).

**Table 3:** Age-adjusted partial correlations between sAGEP and FPG, 2h-PG, and HbA1c.

	sAGEP			
	NGT	IGR	Diabetes	Overall
FPG	0.21 (<0.001)	0.26 (<0.001)	0.49 (<0.001)	0.64 (<0.001)
2h-PG	0.18 (<0.001)	-0.05 (0.45)	0.64 (<0.001)	0.75 (<0.001)
HbA1c	0.47 (<0.001)	0.66 (<0.001)	0.92 (<0.001)	0.90 (<0.001)

sAGEP, serum advanced glycation end products-peptides; FPG, fasting plasma glucose; 2h-PG, 2h-plasma glucose; HbA1c, glycated haemoglobin A1c; NGT, normal glucose tolerance; IGR, impaired glucose regulation

Data are presented as *r (P)*.

**Fig 2:**
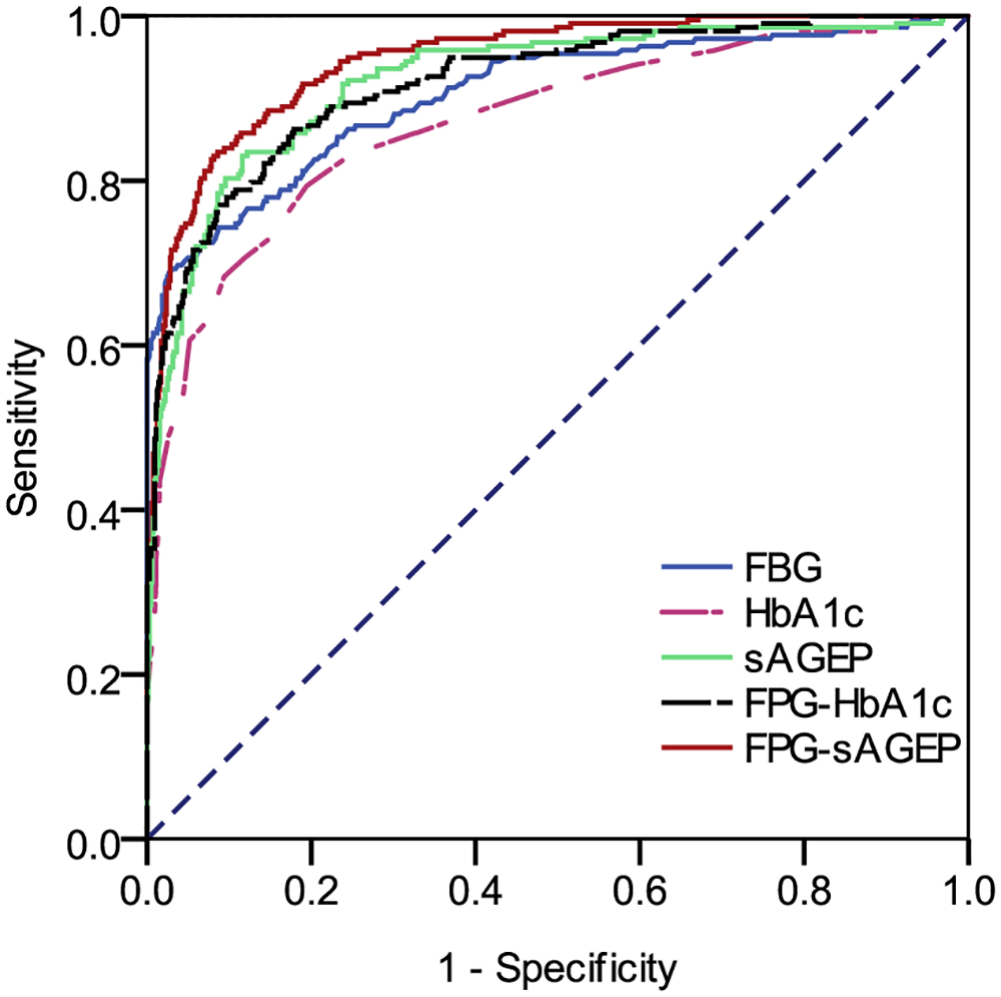
The ROC curves generated by the logistic regression models on FPG, HbA1c, sAGEP, FPG-HbA1c and FPG-sAGEP. ROC, receiver operating characteristic; FPG, fasting plasma glucose; HbA1c, glycated haemoglobin A1c; sAGEP, serum advanced glycation end products-peptides; FPG-HbA1c, FPG plus HbA1c; FPG-sAGEP, FPG plus sAGEP. The areas under the ROC curves for FPG, HbA1c, sAGEP, FPG-HbA1c and FPG-sAGEP were 0.772, 0.838, 0.894, 0.845, and 0.899, respectively. Comparisons among these areas were as follows: FPG-sAGEP vs. FPG-HbA1c (*P* = 0.0003), FPG-sAGEP vs. sAGEP (*P* = 0.58), FPG-sAGEP vs. HbA1c (*P* <0.001), FPG-sAGEP vs. FPG (*P* <0.001), FPG-HbA1c vs. FPG (*P* <0.001), sAGEP vs. HbA1c (*P* = 0.006), sAGEP vs. FPG (*P* <0.001).

The ROC curves generated from the 5 models described in the statistical analysis section and the areas under these ROC curves are shown in Fig 2. Among them, the area under the ROC curve generated from the model on FPG-sAGEP was the largest, while it is larger but non-significantly larger than that on sAGEP (0.899 versus 0.844, *P* = 0.58). The area on FPG-sAGEP is significantly larger than that on FPG-HbA1c (0.899 versus 0.845, *P* = 0.0003), HbA1c (0.899 versus 0.838, *P* <0.001), or FPG (0.899 versus 0.772, *P* <0.001). Given these, the model on FPG-sAGEP was considered the best model in diabetes screening. By using the logistic regression model and maximizing the sum of respective sensitivities and specificities for FPG-sAGEP, the optimal critical line was determined as follows:

0.69 × FPG + 0.14 × sAGEP = 7.03

This optimal critical line yielded a 91.2% sensitivity and a 75.1% specificity in detecting 2h-PG ≥11.1 mmol/L among subjects with FPG <7.0 mmol/L (S1 Table). The critical sensitivity of FPG-sAGEP was significantly higher than that of FPG-HbA1c (91.2% versus 73.6%, *P* <0.001) and HbA1c (91.2% versus 76.9%, *P* <0.001), which alternatively, showed absolute sensitivity advantages of 19.3% over FPG-HbA1c and 15.7% over HbA1c. However, the critical sensitivity was non-significantly higher than that of sAGEP (91.2% versus 89.0%, *P* = 0.62) or FPG (91.2% versus 86.8%, *P* = 0.35). The critical specificity of FPG-sAGEP was significantly lower than that of FPG-HbA1c (75.1% versus 82.9%, *P* <0.001), but was comparable to that of HbA1c or sAGEP.

## Discussion

The results of this study show for the first time that among Chinese subjects with risk factors for diabetes and FPG <7.0 mmol/L, the model on FPG-sAGEP was the best one for diabetes screening to detect a 2h-PG ≥11.1 mmol/L. It had the largest diagnostic accuracy and the highest sensitivity compared with the model on FPG-HbA1c, sAGEP, HbA1c or FPG. Given the evidence that diabetes is diagnosed with FPG ≥7.0 mmol/L and less than half of the new diabetes cases could be detected using this WHO FPG criterion, the model on FPG-sAGEP therefore greatly improved efficacy of the WHO FPG criterion in diabetes screening in this overall population.

Although FPG possesses competitive advantages in terms of its convenience and cost as a preferred test for diabetes screening, the somehow low sensitivity yielded from the cutoff point of FPG at 7.0 mmol/L (the WHO criterion) in detecting new diabetes has greatly questioned its efficacy in screening [17]. In the present study, a sensitivity of 58.3% was found by using this criterion for screening, which is consistent with several other cross-sectional studies [11,34]. As negative screening results are not always timely or directly subject to confirmatory test, the single use of FPG test would cause a latent problem and might contribute to the growing number of undiagnosed diabetes cases, especially among subjects who are at risk for diabetes and have FPG <7.0 mmol/L.

Interestingly, the study by Wang *et al*. showed that HbA1c could significantly improve the efficacy of using FPG alone in diabetes screening [17]. In their work the area generated by the logistic regression model on FPG-HbA1c was significantly larger than that on FPG, and the optimal critical line of FPG-HbA1c gave a critical sensitivity of 58.82% in identifying diabetes in the IFG subjects. Partly in support of these, our findings show that the area generated by the model on FPG-HbA1c was significantly larger than that on FPG (0.845 versus 0.772, *P* <0.001), while the critical sensitivity for this model was 73.6%, a value that is higher than that from Wang *et al*.. It seems likely that different ethnics and target populations involved might contribute to the differences in sensitivities.

Notably, our study shows that the area generated by the model on FPG-sAGEP was significantly larger compared with that on FPG-HbA1c or HbA1c, and the derived optimal critical line of FPG-sAGEP yielded an absolutely higher sensitivity. These findings therefore suggest that sAGEP outperforms HbA1c in improving the efficacy of using FPG alone in diabetes screening among the high-risk Chinese subjects with FPG <7.0 mmol/L, although a lower specificity of FPG-sAGEP than FPG-HbA1c was observed.

The high efficacy of FPG-sAGEP in detecting new cases of diabetes corresponds well with the pathogenesis of abnormal glucose regulation, where excessive postprandial glucose levels would accelerate the accumulations of AGEs in cells and tissues [21,35], while the FPG might remain normal or high but less than 7.0 mmol/L. Moreover, emerging evidence has suggested that AGEs are also markers of oxidative stress, since AGEs increase the formation of reactive oxygen species and impair the antioxidant systems [36,37], leading to insulin resistance and the development of diabetes eventually [38]. Meanwhile, the derived peptides from AGEs were found to be correlated closely with 2h-PG and HbA1c in our study. Given these, it is logical that the combination use of FPG and sAGEP would show great efficacy in diabetes screening. However, it is noteworthy that FPG-sAGEP might be not suitable or sensitive enough for detecting IGR (that is, pre-diabetes), because sAGEP were poorly correlated with 2h-PG in this population.

Since there still exist difficulties in the HbA1c assay standardization in China, and given the evidence above, it sounds reasonable to recommend the optimal critical line generated by the model on FPG-sAGEP for diabetes screening among the high-risk subjects having FPG <7.0 mmol/L. Yet it is worth noting that for those subjects whose FPG and sAGEP are below the optimal critical line but have IFG, an additional OGTT would be recommended for a final conformation, although results from our study suggested that this was unnecessary as the probability for these IFG subjects turned out to be diabetes was zero.

There are several limitations that should be noticed in this study: first, the sample size is relatively inadequate, which may limit the comprehensive interpretation of our findings, although the sample size estimation based on the method described by Schatzkin *et al*. indicates that an abnormality in 80 subjects (with diabetes but FPG <7.0 mmol/L) is adequate to show a superior sensitivity at 80% power with 95% confidence intervals [39]. Second, since the clearance of sAGEP is mainly mediated by kidney and is markedly impaired in patients with chronic kidney disease [40], this alternative and efficient screening approach might be therefore inappropriate for such population. Although subjects with chronic kidney disease or recent histories of acute kidney injury had already been excluded from our study initially, it cannot be ruled out the possibility that some cases who had high sAGEP concentrations probably resulting from incident kidney disease at entry might be diagnosed with diabetes, while they had NGT or IGR in actual. However, the specificities of the models on FPG-sAGEP and sAGEP, which were higher than 75% and comparable to that of HbA1c, suggest that the misdiagnosis rates (false positive rates) are relatively low, fostering confidence in the recommendation of using sAGEP for diabetes screening. Third, it should be acknowledged that the constituents of AGEP were not identified in our study, since they are a mixture of the small-sized peptides from AGEs [22]. However, it is speculated that both Nϵ-(carboxymethyl) lysine (CML) and non-CML AGEs coexist in circulating AGEP, as noted by Takeuchi *et al*. [41]. Moreover, the concentrations of sAGEP determined by fluorescence spectroscopy, a method that is also applied widely for measuring AGEs concentrations [42,43], could not represent the whole ones, given the evidence that peptides from some AGEs such as CML, pyrraline and imidazolone are nonfluorescent [42]. Fourth, despite of an impressive diagnostic accuracy and a high sensitivity of FPG-sAGEP in diabetes screening, the measurement assay for sAGEP requires the use of a flow injection assay system, which might be unavailable in some regions, raising the concern regarding their wide use. Besides, the employment of HPLC in this study makes the procedures of measuring sAGEP somehow complex and thereby increases the cost. However, it should be mentioned that the chromatographic column, a core component of HPLC equipment, was not used in this flow injection assay system, making the cost of measuring AGEP much cheaper than that of HbA1c. Fifth, although the methods for preparation of AGEs and AGEP calibrators are well-established and well-standardized [22,42], there is still the possibility that these *in vitro* prepared calibrators might be different to those using clinical samples. This would potentially weaken the robustness of our current findings. Finally, because our study only involved Chinese Han ethnic subjects with high risk factors for diabetes, it remains largely unknown whether this diabetes screening approach would also be effective in other populations with different ethnics or from different countries. Furthermore, although age has been corrected for in some data analyses, the robustness of our main findings is still likely to be weakened, given the fact that aging accelerates AGE accumulation [44] while our study failed to perform analyses (e.g., the ROC curve, the optimal critical line) in age-stratified groups because of the relatively small sample size.

In light of the current evidence and limitations from this study, future studies with larger sample sizes, enrolling age-stratified populations with risk factors for diabetes and different ethnics are required to further validate the FPG-sAGEP screening approach. Moreover, studies aiming to identify the constituents of AGEP as well as to simplify the measurement assay for AGEP (e.g., using an alternative instrument other than HPLC) are also in need, which will surely help to facilitate the wide use of FPG-sAGEP for screening. Additionally, it has been well documented that AGEs contribute to a variety of microvascular and macrovascular complications of diabetes including diabetic retinopathy and cardiovascular disease [26,35], studies that investigate whether AGEP could be applied as biomarkers for screening for these complications are therefore worth being conducted.

## Conclusions

In conclusion, although FPG is easy to access and reproducible, more than half of the new cases of diabetes are left undetected among high-risk Chinese subjects using the WHO FPG criterion (FPG ≥7.0 mmol/L). The model on FPG-sAGEP substantially improve the efficacy of using FPG alone in diabetes screening among high-risk Chinese subjects having FPG <7.0 mmol/L. Its large accuracy and high sensitivity in detecting diabetes, which are superior to those of FPG-HbA1c, HbA1c, sAGEP, or FPG, making it well suited for opportunistic diabetes screening. Future studies with large sample sizes that employ this proposed approach for diabetes screening among age–stratified, high-risk populations with different ethnics and FPG <7.0 mmol/L are required. However, the optimal critical line of FPG-sAGEP might then require some adjustments prior to its use.

## Supporting Information

S1 Fig Calibration curve and measurement record. (DOCX)

S1 Table Parameters related to the optimal critical line from the model on the fasting plasma glucose plus serum advanced glycation end products-peptides. (DOCX)
