# Supplementary material for: Using Serum Advanced Glycation End Products-Peptides to Improve the Efficacy of World Health Organization Fasting Plasma Glucose Criterion in Screening for Diabetes in High-Risk Chinese Subjects
Source: PLoS One. 2015 Sep 14;10(9):e0137756. doi: 10.1371/journal.pone.0137756 (PMC4569373; doi:10.1371/journal.pone.0137756)
Supplement: S1 Fig — (DOCX) [file pone.0137756.s001.docx]

(A).


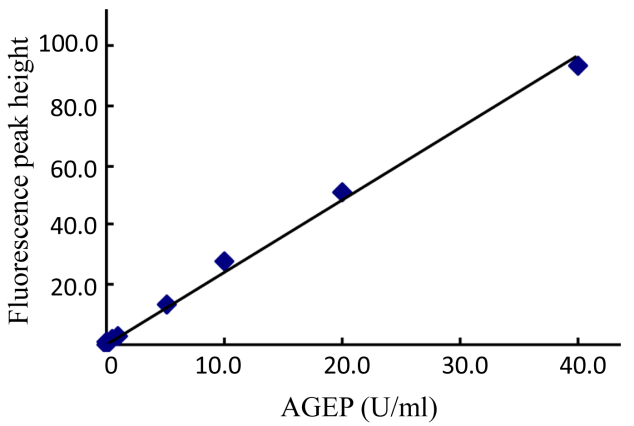


(B)


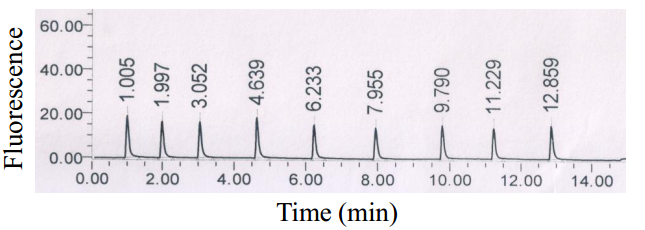


S1 Fig. Calibration curve and measurement record.

(A) The calibration curve for determining AGEP concentrations in unknown serum samples. The equation is y = 1.47 + 2.34x, where y is the value of the average fluorescence peak height, and x is the value of AGEP concentrations. The blue square represents the average fluorescence peak height from each standard AGEP sample assayed in triplicate together with their corresponding concentrations. (B) The original measurement record for 3 serum samples assayed in triplicate using the proposed flow injection system. AGEP, advanced glycation end products-peptides
