# Supplementary material for: Using Serum Advanced Glycation End Products-Peptides to Improve the Efficacy of World Health Organization Fasting Plasma Glucose Criterion in Screening for Diabetes in High-Risk Chinese Subjects
Source: PLoS One. 2015 Sep 14;10(9):e0137756. doi: 10.1371/journal.pone.0137756 (PMC4569373; doi:10.1371/journal.pone.0137756)
Supplement: S1 Table — (DOCX) [file pone.0137756.s002.docx]

# S1 Table. Parameters related to the optimal critical line from the model on the fasting plasma glucose plus serum advanced glycation end products-peptides.

| Parameters A | Coefficient | Standard Error | *P* |
| --- | --- | --- | --- |
| b | 0.67 | 0.22 | 0.002 |
| c | 0.14 | 0.02 | <0.001 |
| a | -9.39 | 1.20 | <0.001 |

In parameters A, a, b, and c were the estimated intercept, coefficient for fasting plasma glucose, and coefficient for serum advanced glycation end products-peptides, respectively.

Results for these parameters were derived from the logistic regression analysis.

| Parameters B | Sensitivity  (%) | Specificity  (%) |  |
| --- | --- | --- | --- |
| R |  |  |  |
| 0.0693 | 93.4 | 6.72 |  |
| 0.0740 | 92.3 | 69.0 |  |
| 0.0763 | 91.2 | 70.1 |  |
| 0.0799 | 91.2 | 71.3 |  |
| 0.0825 | 91.2 | 72.6 |  |
| 0.0845 | 91.2 | 73.8 |  |
| 0.0864 | 91.2 | 75.1 | k was achieved here |
| 0.0903 | 90.1 | 75.9 |  |
| 0.0934 | 86.8 | 76.2 |  |
| 0.0953 | 86.8 | 76.8 |  |
| 0.102 | 85.7 | 78.1 |  |
| 0.105 | 84.6 | 79.5 |  |
| 0.110 | 82.4 | 80.1 |  |

In parameters B, R was the cost-to-benefit ratio, where k was the optimal cutoff point determined by maximizing the sum of sensitivity and specificity. From this table, k was determined as 0.0864.

Based on these parameters, the optimal critical line from the model on the fasting plasma glucose plus serum advanced glycation end products-peptides would be 0.69×FPG+0.14×sAGEP=7.03.
